# *Ca*. Nitrososphaera and *Bradyrhizobium* are inversely correlated and related to agricultural practices in long-term field experiments

**DOI:** 10.3389/fmicb.2013.00104

**Published:** 2013-05-01

**Authors:** Kateryna Zhalnina, Patricia D. de Quadros, Kelsey A. Gano, Austin Davis-Richardson, Jennie R. Fagen, Christopher T. Brown, Adriana Giongo, Jennifer C. Drew, Luis A. Sayavedra-Soto, Dan J. Arp, Flavio A. O. Camargo, Samira H. Daroub, Ian M. Clark, Steve P. McGrath, Penny R. Hirsch, Eric W. Triplett

**Affiliations:** ^1^Department of Microbiology and Cell Science, Institute of Food and Agricultural Sciences, University of FloridaGainesville, FL, USA; ^2^Department of Soil Science, Federal University of Rio Grande do SulPorto Alegre, Brazil; ^3^Department of Botany and Plant Pathology, Oregon State UniversityCorvallis, OR, USA; ^4^Everglades Research and Education Center, University of FloridaBelle Glade, FL, USA; ^5^Rothamsted ResearchHarpenden, Hertfordshire, UK

**Keywords:** agricultural land use, ammonia-oxidizing archaea, diazotrophs, *Ca*. Nitrososphaera, *Bradyrhizobium*, soil properties

## Abstract

Agricultural land management, such as fertilization, liming, and tillage affects soil properties, including pH, organic matter content, nitrification rates, and the microbial community. Three different study sites were used to identify microorganisms that correlate with agricultural land use and to determine which factors regulate the relative abundance of the microbial signatures of the agricultural land-use. The three sites included in this study are the Broadbalk Experiment at Rothamsted Research, UK, the Everglades Agricultural Area, Florida, USA, and the Kellogg Biological Station, Michigan, USA. The effects of agricultural management on the abundance and diversity of bacteria and archaea were determined using high throughput, barcoded 16S rRNA sequencing. In addition, the relative abundance of these organisms was correlated with soil features. Two groups of microorganisms involved in nitrogen cycle were highly correlated with land use at all three sites. The ammonia oxidizing-archaea, dominated by *Ca*. Nitrososphaera, were positively correlated with agriculture while a ubiquitous group of soil bacteria closely related to the diazotrophic symbiont, *Bradyrhizobium*, was negatively correlated with agricultural management. Analysis of successional plots showed that the abundance of ammonia oxidizing-archaea declined and the abundance of bradyrhizobia increased with time away from agriculture. This observation suggests that the effect of agriculture on the relative abundance of these genera is reversible. Soil pH and NH_3_ concentrations were positively correlated with archaeal abundance but negatively correlated with the abundance of *Bradyrhizobium*. The high correlations of *Ca.* Nitrososphaera and *Bradyrhizobium* abundances with agricultural management at three long-term experiments with different edaphoclimatic conditions allowed us to suggest these two genera as signature microorganisms for agricultural land use.

## Introduction

The transformation of soil to agricultural use causes significant changes in its chemical, physical, and biological features, including changes in the microbial community composition (Kibblewhite et al., 2008). A number of studies have examined the impact of agriculture on microbial community structure. The effect of inorganic and organic fertilizers, tillage, and different crop rotations was analyzed. It was found that nitrogen (N) deposition increases the abundance of certain microbial phyla, such as Actinobacteria, Proteobacteria, Bacteroidetes, and Firmicutes, but it also decreases the abundance of other bacterial phyla, such as Acidobacteria and Verrucomicrobia (Ramirez et al., 2010, 2012; Fierer et al., 2012). An increase in microbial biomass was detected after farmyard manure addition (Kandeler et al., 1999), while previous studies have shown that reducing tillage increased microbial biomass (Buckley and Schmidt, 2001; Plassart et al., 2008). Other studies have focused on the changes of specific microbial taxa involved in nutrient cycling in soil, such as diazotrophs (Meng et al., 2012), nitrifiers (Chu et al., 2008), and denitrifiers (Clark et al., 2012) under specific agricultural management. Therefore, all studies of the agricultural impact on the microbial community are limited either to the phylum level, which represents a large and diverse set of functional microbial groups, or to the specific physiological group. We hypothesize that there are common microbial taxa strongly associated with agricultural land use, and these taxa can be used as indicators to monitor the level of the land disturbance during agricultural management.

One of the abundant groups found in many soils under agricultural use is ammonia-oxidizing archaea (AOA) (He et al., 2007; Shen et al., 2008; Gubry-Rangin et al., 2010; Taketani and Tsai, 2010; Wessén et al., 2010; Pratscher et al., 2011; Xia et al., 2011). In soils such as Amazonian Anthrosol, agricultural plots have a higher archaeal ammonia monooxygenase gene (*amoA*) copy number than in adjacent soils (Taketani and Tsai, 2010). Wessén et al. (2011) showed that high numbers of archaea are positively correlated with nitrate leaching from both integrated and organic farming systems. Two agricultural soils in Germany, OrthicLuvisol and GleyicCambisol, harbored high abundances of archaeal, but not bacterial *amoA* genes (Schauss et al., 2009). AOA are important participants in soil nitrification because they are able to perform the first step of nitrification through the ammonia monooxygenase enzyme (AMO) (Könneke et al., 2005; Treusch et al., 2005). The most represented soil AOA was found within was *Candidatus* Nitrososphaera (Taketani and Tsai, 2010; Xia et al., 2011; Pester et al., 2012).

Agricultural management intensifies all processes related to the N cycle including an increase in nitrification rates (Lu et al., 2011). Increased nitrification can result in nitrate leaching into the surface and ground water, as well as the emission of nitrous oxide to the atmosphere (Kim et al., 2012). Recently, the contribution of AOA to nitrification was shown by assimilation of ^13^C-CO_2_ by AOA during soil nitrification (Zhang et al., 2010, 2012; Xia et al., 2011). In addition a high correlation between AOA and nitrification activity was observed (Offre et al., 2009) as well as high abundance of archaeal *amoA* transcripts in soil (Treusch et al., 2005; Nicol et al., 2008). Hence, AOA may be responsible for all these consequences of intensified nitrification. Knowledge of the factors that may drive the abundance of this group is necessary to prevent agricultural land management from negatively impacting the environment. Previous studies have investigated temperature, soil type, and elevation as drivers of archaeal abundance in soils (Zhang et al., 2009; Taketani and Tsai, 2010). Other studies have examined pH, fertilization, carbon to nitrogen ratio, and tillage as anthropogenic drivers that are a result of land management (Kandeler et al., 1999; Fierer and Jackson, 2006; Enwall et al., 2007; Nicol et al., 2008; Taketani and Tsai, 2010; Bates et al., 2011; Hermansson and Lindgren, 2001). Gubry-Rangin et al. (2011) found different phylogenetic lineages of AOA that were acidophilic, acido-neutrophilic, and alkalinophilic, and these were positively correlated with soil pH levels. Bru et al. (2011) found a positive correlation between AOA abundance and pH. Pereira e Silva et al. (2012) have recently shown that AOA abundance was positively correlated with pH in a temporal study based on eight agricultural soils. However, Nicol et al. (2008) reported results where archaeal *amoA* gene and transcript abundance decreased with higher pH in acidic soils. The factors responsible for the changes in AOA abundance vary depending on the ecosystem, the geography, and soil type. It remains unclear what factors are mainly responsible for triggering the abundance of archaea in soil.

Three long-term experiments that study crop production, nutrient cycling, and environmental impact of agriculture were included in this work. The first experiment, the Broadbalk Rothamsted Experiment, was designed more than 170 years ago to test the effects of various combinations of inorganic fertilizers and farmyard manure on the yield of the wheat. The second study site, the Kellogg Biological Station long-term ecological research project, was initiated to examine the basic ecological relationships in field-crop ecosystems typical of the Midwestern U. S. Early in the last century, 280,000 ha of primary rich with organic matter histosols in South Florida were drained to create the Everglades Agricultural Area, which was the third experiment for our study. About 25% of US winter vegetables and sugarcane are cultured in the EAA. Despite the many years of study at the Broadbalk Rothamsted Experiment, the Everglades Agricultural Area, and the Kellogg Biological Station, there are few studies describing how agricultural management practices affect microbial taxa at these long-term sites (Castro et al., 2005; Ogilvie et al., 2008; Ramirez et al., 2010; Delmont et al., 2011; Clark et al., 2012; Fierer et al., 2012). None of these show the detailed differences in taxonomic groups that occur with agricultural land use and none of them show this across multiple sites. Also, none of these studies provide evidence for biomarkers of land use or show how microbial taxa change with land use succession. The goals of this study were to examine changes in archaeal and bacterial community composition in response to land-use with a particular emphasis on ammonia oxidizers. Microbial communities in the soils of three long-term agricultural sites were examined using 16S rRNA barcoded Illumina sequencing. Sites were also chosen so that the effect of succession on microbial taxa could be examined. Soil community composition was also compared to several soil properties to identify the drivers of microbial diversity and abundance.

## Materials and methods

### Study sites

Soil samples from agricultural and non-agricultural areas of three different long-term experimental sites were collected for this work:

- *Broadbalk Rothamsted Research (BRR)*—Located at Rothamsted Research, Harpenden, UK, the BRR soil is an Alfisol flinty-silty clay loam. BRR is the oldest long-term agronomic experiment in the world. Apart from occasional fallowing, the arable management plots have been in continuous winter wheat for 168 years. Nitrogen is added as farmyard manure (FYM) at 35 t/ha in autumn and/or as ammonium nitrate in spring ranging from 0 to 288 kg ha^−1^ per year. The BRR experiment included ten agricultural treatments: six N treatments (0, 48, 96, 144, 192, 288 kg ha^−1^ per year); three FYM with or without additional N (N: 0, 96, 192 kg ha^−1^ per year); and one is the nil application treatment of no fertilizers or organic amendments. Two non-agricultural treatments include unfertilized grassland or woodland. All treatments had three pseudoreplicates and were sampled monthly over 5-month period (May to September) (Goulding et al., 2000; Poulton, 2012).
- *Michigan Kellogg Biological Station (KBS)*—Located at the Michigan Kellogg Biological Station (KBS), Kalamazoo, Michigan, USA, the KBS Alfisol soil site has plots planted to a corn-soybean-wheat rotations under conventional till system since 1980. Ammonium nitrate is added three times during the growing season, beginning in April and ending in November. Rates of N application range from 153 to 165 kg ha^−1^ per year for corn and from 56 to 90 kg ha^−1^ per year for wheat. At KBS, 15 samples were collected from agricultural plots (five biological replicates with three pseudoreplicates of each were collected from T1 agricultural plots under conventional tillage with a rotation of corn, soybean, and wheat), and nine samples were collected from non-agricultural plots (SF1, SF2, SF3 plots with three pseudoreplicates of each). The non-agricultural plots SF2 (Louden Field) and SF3 (Turner Field) are successional old fields abandoned from cropping in 1951 and 1963, respectively. Plot SF1, Canton Field, was last managed as an agricultural system in 1971 (Robertson et al., 1993).
- *Everglades Agricultural Area (EAA)*—Located near Belle Glade, Florida, USA, the EAA Histosol contains rich organic soils overlying limestone. In the early 1900s, the Everglades region began to be drained for agricultural purposes for winter vegetables and sugarcane production. Drainage increased the level of oxygen in soils and created conditions favorable for aerobic microorganisms that decompose soil organic matter (SOM). Since the draining, decomposition of SOM in EAA soils exceed their accumulation resulting in subsidence of Everglades soils at the annual rate of about 15–25 mm (Snyder, 2005). Mineralization of organic nitrogen occurs at higher levels than is required for crops, resulting in drainage water contamination (Bottcher and Izuno, 1994). Three replicates each of the agricultural sugarcane plots, SR1, SR2, and SR3, and the non-agricultural virgin plots, VR1, VR2, and VR3, were sampled at the EAA monthly over a 1-year period.

### Soil sampling

At all sites, soil sampling was collected in three replicates: 3 cm diameter corers, pre-washed with 70% ethanol, were inserted into the soil to a depth of 10 cm. For each replicate, 10 cores were pooled and samples were then sieved through a 2 mm sieve and thoroughly mixed. Each replicate was then frozen at −80°C for subsequent DNA extraction.

### Analysis of soil parameters

For all sites, soil parameters including pH, percent moisture, total N, NO_3_-N and NH^+^_4_-N were measured (Walkley and Black, 1934; Schofield and Taylor, 1955; Black, 1965; Mulvaney, 1996; Ronghong and Lawrence, 2010). NH_3_ levels were calculated from the soil NH^+^_4_-N concentrations using the pKa of NH_3_ (9.23) and soil pH. Three measurements were made for each sampling plot and then averaged to give a representative value. Soil pH was measured using a glass electrode in 1:2 suspension of soil in dH_2_O (Schofield and Taylor, 1955). Gravimetric water content (soil moisture) was determined as gravimetric water content by drying 10 g soil at 105°C for 24 h (Black, 1965). At BRR, total N and C were determined using the combustion method (LECO CNS 2000). NO^−^_3_-N and NH^+^_4_-N were extracted with 2 M KCl for 2 h. After extraction, the supernatant was filtered through Whatman No. 1 filter paper and the supernatant was analyzed for NH^+^_4_ and NO^−^_3_-N by an automated colorimetric assay (Skalar SAN^PLUS^ System; Skalar, Breda, The Netherlands). At EAA and KBS, total N, NO^−^_3_-N and NH^+^_4_-N were measured according to previously described protocols (Mulvaney, 1996; Ronghong and Lawrence, 2010). At BRR and KBS the % soil organic carbon was determined by the Walkley-Black chromic acid wet oxidation method (Walkley and Black, 1934). Soil organic matter (SOM) was calculated by multiplying % organic carbon by a factor of 1.72.

### DNA extraction

For each sample, DNA was isolated from 0.25 g of soil using the MoBioPowerSoil™ DNA Isolation Kit (Carlsbad, CA, USA). Extractions were performed according to the manufacturer's protocol for samples collected from EAA and KBS. Samples from Broadbalk were extracted as described by the manufacturer except for the use of the MP Biomedicals FastPrep-24 machine for 30 s at 5.5 m/s, instead of vortex agitation. All genomic DNA concentration and purity was determined by NanoDrop spectrophotometry (Thermo Scientific, Wilmington, DE, USA).

### Illumina high-throughput sequencing of 16S rRNA genes and taxonomic classification of sequence reads

Bacterial and archaeal 16S rRNA genes were amplified using barcoded universal prokaryotic primers515F (5′-GTGCCAGCAGCCGCGGTAA-3′) and 806R (5′-GGACTACVSGGGTATCTAAT-3′) (Caporaso et al., 2010) and sequenced using Illumina technology as described previously (Fagen et al., 2012). Classification of reads was done using previous methods (Giongo et al., 2010a,b) modified to the paired-end Illumina platform (Fagen et al., 2012). Reads were trimmed to remove low quality bases and to remove the first 11 bases corresponding to the primer region by a script based on Trim2 (Huang et al., 2003, source available at: https://gist.github.com/1006830), and then the reads were separated by barcode (source available at: https://gist.github.com/1006983). This resulted in 11,390,227, 1,307,720, and 1,739,319 trimmed reads from BRR, KBS, and EAA, respectively, with an average read length of 158 bases. Paired reads were assembled using CLC Assembly Cell v3.0.2b to the reference Ribosomal Database Project (RDP) (Cole et al., 2009) 16S SSU rRNA database. Full taxonomic descriptions based on the NCBI taxonomy database (http://www.ncbi.nlm.nih.gov) were generated for the entries in the RDP database using TaxCollector (Giongo et al., 2010b). Matches were filtered at 80% length fraction and classified at the 80% identity level for domain and phylum, 90% identify level for class, order and family, 95% identity level for genus, and 99% identity level for species. The total number of pairs matching 16S rRNA sequences in the database at each level of similarity created an OTU abundance matrix for each level of taxonomy across samples. Pairs that did not match to the same sequence in the RDP database were annotated according to their Last Common Ancestor (LCA), and pairs that did not have an LCA, or any match in the RDP database, were considered to be unclassified. To normalize for varying sequencing depths, the OTU abundance matrices for each sample were divided by the total number of pairs after trimming.

### Statistical analysis

Statistical analysis was performed using the R statistical package (R Development Core Team, 2011) and XLSTAT-Pro 2011. Spearman correlations (using *p* ≤ 0.001) for non-normally distributed data were used to independently evaluate the correlation of each measured soil parameter with the relative abundance of taxa. For ANOVA, the relative abundance of taxa was transformed to the arcsine square root to satisfy the normality assumption. One-Way ANOVAs were used to compare relative abundance of taxa in agricultural and non-agricultural plots at all study sites. A Two-Way ANOVA was used to determine if agriculture and study site had a significant effect on the relative abundance of taxa.

## Results

### Sequence analysis of 16S rRNA genes and microbial community at agricultural and non-agricultural sites

The total number of barcoded reads obtained from sequencing ranged between 1.3 and 11.4 million reads with an average length of 158 bp (Table 1). 94.3%, 95.5%, and 91.1% sequences were classified as *Bacteria*; 3%, 1.3%, and 3.7% sequences as *Archaea* at BRR, KBS, and EAA, respectively. The taxonomic classification of the ten most abundant genera from each of the three study sites includes 19 different genera in total (Table 2). Eight genera were from the Proteobacteria (five alpha, two gamma, and one beta), four from the Actinobacteria, three from the Bacteroidetes, two from the Firmicutes, and one genus each from the Acidobacteria and Thaumarchaeota. *Ca*. Nitrososphaera and *Pseudomonas* were the most abundant genera at Broadbalk. For the Everglades, the most abundant genera were *Ca*. Nitrososphaera and *Rhodoplanes*, and at the Kellogg Biological Station, the most abundant genera were *Ca*. Nitrososphaera and *Sphingomonas*.

**Table 1:** **Results of Illumina sequencing**.

**Site**	**Total number of reads**	**Average of paired reads per sample**	**Number of operational taxonomic units**				
			**Phylum**	**Class**	**Order**	**Family**	**Genus**
BRR	11,390,227	64,717	24	40	92	232	1021
KBS	1,307,720	31,136	25	39	84	201	741
EAA	1,739,319	23,191	25	41	90	217	860

Number of Illumina sequencing reads at five taxonomic levels from Rothamsted Research (BRR), the Everglades Agricultural Area (EAA) and the Kellogg Biological Station (KBS).

**Table 2:** **The 10 most abundant genera found in soils at three experimental sites at agricultural and non-agricultural plots**.

**Genus**	**BRR**				**KBS**				**EAA**			
	**Ag**	**Non-ag**	**Average**	**Stdev**	**Ag**	**Non-ag**	**Average**	**Stdev**	**Ag**	**Non-ag**	**Average**	**Stdev**
*Ca.* Nitrososphaera	3.43	0.53	2.92 (1943)	1.45	1.41	0.41	1.04 (323)	0.88	3.56	1.68	2.62 (608)	1.23
*Pseudomonas*	1.48	0.44	1.39 (926)	2.61	0.2	0.23	0.22 (70)	0.20	0.11	0.03	0.06 (14)	0.08
*Bradyrhizobium*	0.78	1.94	0.97 (647)	0.54	0.65	1.33	0.89 (276)	0.44	0.09	0.63	0.35 (80)	0.32
*Sphingomonas*	0.97	0.64	0.92 (609)	0.62	2.43	0.26	1.79 (555)	1.21	0.47	0.41	0.44 (101)	0.20
*Flavobacterium*	0.78	0.72	0.77 (512)	0.72	1.18	0.25	0.20 (63)	0.18	0.35	0.06	0.22 (50)	0.30
*Nocardioides*	0.76	0.47	0.72 (476)	0.37	0.37	0.13	0.29 (90)	0.17	0.31	0.1	0.21 (48)	0.14
*Rhodoplanes*	0.49	1.36	0.63 (417)	0.40	0.53	0.5	0.53 (165)	0.12	0.57	1.37	0.96 (223)	0.46
*Steroidobacter*	0.60	0.71	0.62 (410)	0.15	0.31	0.09	0.24 (74)	0.16	0.17	0.24	0.19 (45)	0.13
*Bacillus*	0.46	1.21	0.59 (394)	0.59	0.72	1.31	0.92 (285)	0.71	0.6	0.45	0.55 (127)	0.46
*Nitrospira*	0.52	0.49	0.52 (347)	0.17	0.4	0.23	0.35 (107)	0.15	0.82	0.7	0.77 (177)	0.34
*Paenibacillus*	0.34	0.99	0.45 (297)	0.29	0.31	0.61	0.42 (129)	0.25	0.17	1.45	0.78 (180)	0.75
*Streptomyces*	0.44	0.53	0.46 (304)	0.19	0.63	0.24	0.52 (162)	0.30	0.05	0.31	0.19 (45)	0.16
*Mycobacterium*	0.31	1.00	0.42 (282)	0.30	0.43	0.71	0.54 (166)	0.25	0.02	0.18	0.1 (23)	0.11
*Arthrobacter*	0.44	0.10	0.38 (255)	0.26	0.25	0.08	0.20 (62)	0.11	0.66	0.01	0.33 (77)	0.36
*Hyphomicrobium*	0.36	0.39	0.36 (242)	0.12	0.22	0.14	0.19 (61)	0.08	0.38	0.13	0.27 (62)	0.15
*Terrimonas*	0.32	0.08	0.28 (189)	0.20	0.33	0.15	0.27 (83)	0.19	0.39	0.27	0.31 (71)	0.13
*Flavisolibacter*	0.15	0.02	0.13 (85)	0.13	0.77	0.12	0.54 (168)	0.43	0.21	0.13	0.17( 39)	0.11
*Burkholderia*	0.04	0.12	0.06 (37)	0.04	0.84	0.57	0.78 (243)	0.45	0.09	0.26	0.17 (38)	0.14
*Ca.* Koribacter	0.02	0.0	0.02 (12)	0.01	0.62	0.57	0.62 (191)	0.39	0.0	0.91	0.42 (98)	0.53

Relative abundance represented as a proportion of 16S rRNA gene reads of the total number of reads from each site (%) at agricultural (Ag), non-agricultural (Non-ag) plots, and average proportion of 16S rRNA reads at each experiment. Numbers in the brackets represent average number of reads for each taxon.

**Figure 1:**
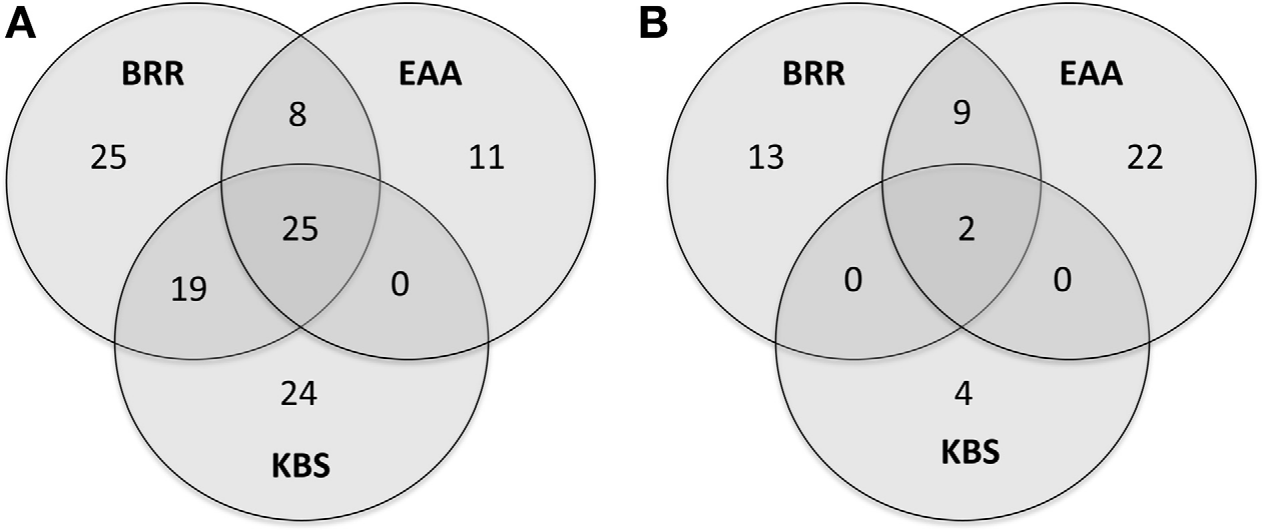
**Venn diagram of the most abundant genera.** Venn diagram showing overlap of the most abundant genera (those with ≥0.05% of all 16S rRNA reads) between soils from three experimental sites at the Broadbalk Experiment at Rothamsted Research (BRR), the Everglades Agricultural Area (EAA) and the Kellogg Biological Station (KBS) **(A)**. Venn diagram showing overlap of the most abundant genera correlated with agricultural management at the three experimental sites (rho ≥ 0.5, *p* ≤ 0.001) **(B)**.

The genera with a relative abundance of at least 0.05% of all total 16S rRNA reads from each site were analyzed for all three sites and represented in a Venn diagram (Figure 1A). Twenty-five genera (13.2%) were common to all three sites.

Correlations between the relative abundance of archaeal and bacterial taxa and agricultural management were calculated across all sites. Only those genera that represented at least 0.05% of total reads across any site were examined. Twenty-seven genera were significantly positively correlated (rho ≥ 0.5, *p* ≤ 0.001) with agricultural use while 23 genera were negatively correlated with agriculture (rho ≤ −0.5, *p* ≤ 0.001, Table 3). Only two genera, *Ca*. Nitrososphaera and *Bradyrhizobium*, showed significant correlations with agricultural management at all three sites (Figure 1B, Table 3). In addition, nine genera were significantly correlated with agriculture at BRR and EAA. *Marmoricola, Blastococcus, Ramlibacter*, and *Lysobacter* were positively correlated with BRR and EAA agricultural management (Table 3), while *Rhodoplanes, Mycobacterium, Paenibacillus*, and *Burkholderia* abundances were significantly higher at non-agricultural plots and negatively correlated with agricultural land-use (Tables 2, 3).

**Table 3:** **Genera highly correlated with agriculture in bold (16S rRNA ≥ 0.05%, rho ≥ ±0.5, *p* ≤ 0.001)**.

**Genus**	**Phylum**	**Spearman correlation (rho value)**								
		**Agricultural**			**pH**			**NH**_**3**_		
		**BRR**	**EAA**	**KBS**	**BRR**	**EAA**	**KBS**	**BRR**	**EAA**	**KBS**
*Ignavibacterium*	Chlorobi	−	**0.87**	−	−	**0.85**	−	−	**0.67**	−
*Marmoricola*	Actinobacteria	**0.51**	**0.85**	−	0.09	**0.76**	−	0.03	**0.61**	−
*Arthrobacter*	Actinobacteria	**0.58**	**0.85**	−	0.19	**0.74**	−	0.20	**0.65**	−
*Ca.* Entotheonella	Proteobacteria	−	**0.84**	−	−	**0.83**	−	−	**0.62**	−
*Adhaeribacter*	Bacteoidetes	−	**0.83**	−	−	**0.76**	−	−	**0.55**	−
*Blastococcus*	Actinobacteria	**0.62**	**0.83**	−	**0.48**	**0.74**	−	**0.37**	**0.66**	−
*Hyphomicrobium*	Proteobacteria	−	**0.80**	−	−	**0.71**	−	−	**0.59**	−
*Ramlibacter*	Proteobacteria	**0.57**	**0.80**	−	0.20	**0.79**	−	−0.06	**0.58**	−
***Ca.* Nitrososphaera**	Thaumarchaeota	**0.63**	**0.78**	**0.70**	**0.44**	**0.73**	0.50	**0.37**	**0.56**	−0.54
*Nocardioides*	Actinobacteria	−	**0.78**	−	−	**0.66**	−	−	**0.48**	−
*Prosthecomicrobium*	Proteobacteria	−	**0.76**	−	−	**0.70**	−	−	**0.62**	−
*Lysobacter*	Proteobacteria	**0.60**	**0.73**	−	**0.51**	**0.71**	−	0.18	**0.47**	−
*Woodsholea*	Proteobacteria	−	**0.71**	−	−	**0.60**	−	−	**0.68**	−
*Pseudomonas*	Proteobacteria	−	**0.57**	−	−	**0.42**	−	−	0.36	−
*Flavobacterium*	Bacteoidetes	−	**0.52**	−	−	**0.54**	−	−	0.35	−
*Antarcticicola*	Proteobacteria	**0.50**	−	−	**0.56**	−	−	**0.42**	−	−
*Azospirillum*	Proteobacteria	**0.55**	−	−	**0.35**	−	−	0.15	−	−
*Cystobacter*	Proteobacteria	**0.55**	−	−	**0.41**	−	−	**0.26**	−	−
*Dechloromonas*	Proteobacteria	**0.51**	−	−	0.10	−	−	−0.12	−	−
*Desulfuromonas*	Proteobacteria	**0.55**	−	−	0.24	−	−	0.13	−	−
*Flavisolibacter*	Bacteroidetes	−	−	**0.84**	−	−	**0.66**	−	−	−0.07
*Luteimonas*	Proteobacteria	**0.56**	−	−	**0.32**	−	−	0.01	−	−
*Methylobacterium*	Proteobacteria	**0.57**	−	−	**0.59**	−	−	**0.39**	−	−
*Nostoc*	Cyanobacteria	**0.59**	−	−	**0.40**	−	−	0.17	−	−
*Skermanella*	Proteobacteria	**0.60**	−	−	**0.47**	−	−	**0.31**	−	−
*Sphingomonas*	Proteobacteria	−	−	**0.84**	−	−	0.61	−	−	−0.06
*Terrimonas*	Bacteroidetes	**0.56**	−	−	**0.42**	−	−	0.07	−	−
*Actinoallomurus*	Actinobacteria	−	**−0.87**	−	−	**−0.83**	−	−	**−0.71**	−
*Ca.* Koribacter	Acidobacteria	−	**−0.87**	−	−	**−0.80**	−	−	**−0.69**	−
*Dokdonella*	Proteobacteria	−	**−0.85**	−	−	**−0.80**	−	−	**−0.69**	−
*Rhodoplanes*	Proteobacteria	**−0.64**	**−0.85**	−	**−0.41**	**−0.82**	−	**−0.25**	**−0.70**	−
*Actinomadura*	Actinobacteria	−	**−0.84**	−	−	**−0.82**	−	−	**−0.66**	−
*Acidiphilium*	Proteobacteria	−	**−0.84**	−	−	**−0.81**	−	−	**−0.71**	−
*Ca.* Solibacter	Acidobacteria	−	**−0.84**	−	−	**−0.75**	−	−	**−0.64**	−
*Rhodocista*	Proteobacteria	−	**−0.84**	−	−	**−0.80**	−	−	**−0.61**	−
*Cupriavidus*	Proteobacteria	−	**−0.84**	−	−	**−0.76**	−	−	**−0.69**	−
*Mycobacterium*	Actinobacteria	**−0.59**	**−0.83**	−	**−0.44**	**−0.82**	−	**−0.29**	**−0.66**	−
*Paucimonas*	Proteobacteria	−	**−0.83**	−	−	**−0.72**	−	−	**−0.69**	−
*Streptomyces*	Actinobacteria	−	**−0.82**	−	−	**−0.75**	−	−	**−0.65**	−
*Paenibacillus*	Firmicutes	**−0.64**	**−0.82**	−	**−0.46**	**−0.78**	−	**−0.29**	**−0.68**	−
***Bradyrhizobium***	Proteobacteria	**−0.63**	**−0.82**	**−0.80**	**−0.74**	**−0.83**	**−0.81**	**−0.54**	**−0.69**	−0.07
*Rhodopseudomonas*	Proteobacteria	−	**−0.74**	−	−	**−0.66**	−	−	**−0.55**	−
*Niastella*	Bacteroidetes	−	**−0.70**	−	−	**−0.63**	−	−	**−0.53**	−
*Burkholderia*	Proteobacteria	**−0.55**	**−0.67**	−	**−0.50**	**−0.64**	−	**−0.48**	**−0.52**	−
*Pedomicrobium*	Proteobacteria	−	**−0.63**	−	−	**−0.70**	−	−	**−0.49**	−
*Spartobacteria*	Verrucomicrobia	−	**−0.62**	−	−	**−0.63**	−	−	**−0.50**	−
*Actinoplanes*	Actinobacteria	**−0.56**	−	−	**−0.31**	−	−	−0.17	−	−
*Kribbella*	Actinobacteria	**−0.58**	−	−	**−0.71**	−	−	**−0.51**	−	−
*Phenylobacterium*	Proteobacteria	**−0.53**	−	−	**−0.64**	−	−	**−0.63**	−	−
*Solirubrobacter*	Actinobacteria	**−0.53**	−	−	**−0.39**	−	−	−0.20	−	−

**Figure 2:**
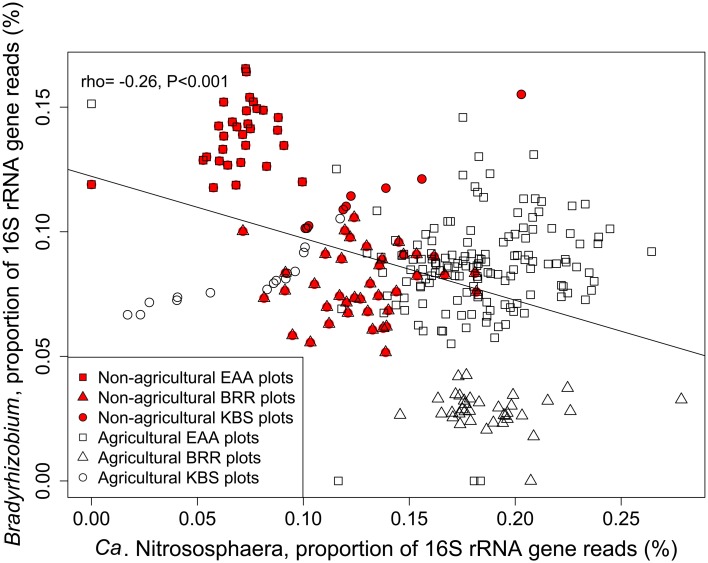
**Proportion of 16S rRNA gene reads of *Ca.* Nitrososphaera and *Bradyrhizobium* at Rothamsted Research (BRR), the Everglades Agricultural Area (EAA) and the Kellogg Biological Station (KBS).** Proportion of 16S rRNA reads was normalized by arcsine square root.

The relative abundances of *Ca*. Nitrososphaera and *Bradyrhizobium* were plotted together to depict the relationship between the relative abundance of these genera and agricultural management (Figure 2). These two genera were inversely correlated with each other (rho = −0.26, *p* ≤ 0.001). Non-agricultural plots exhibited the lower abundance of *Ca.* Nitrososphaera and the higher abundance of *Bradyrhizobium*. Conversely, agricultural plots were represented by the higher abundance of *Ca*. Nitrososphaera and the lower abundance of *Bradyrhizobium*.

**Figure 3:**
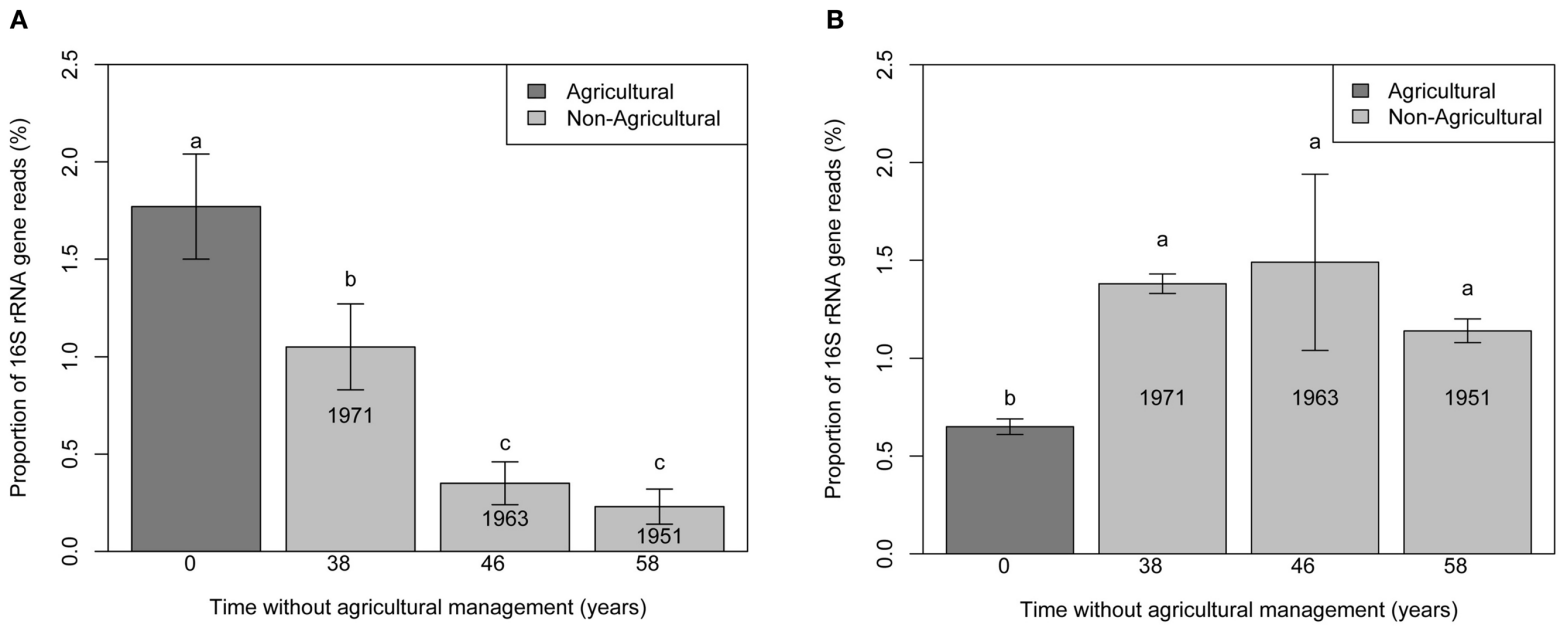
**Bar chart representing the proportion of 16S rRNA gene reads for *Ca.* Nitrososphaera (A) and *Bradyrhizobium* (B) at the Kellogg Biological Station (KBS) site at agricultural plots, and plots without agricultural management.** Error bars indicate standard error. Different letter designations indicate statistically different proportions of microorganism.

In addition, the relative abundances of the 16S rRNA from *Ca.* Nitrososphaera and *Bradyrhizobium* were evaluated at KBS successional plots (Figure 3). The proportion of *Ca.* Nitrososphaera declined steadily with time away from agriculture. In contrast, the proportion of *Bradyrhizobium* increased during the first 38 years without agriculture but remained constant thereafter.

### Ammonia-oxidizing archaea in agricultural and non-agricultural sites

**Figure 4:**
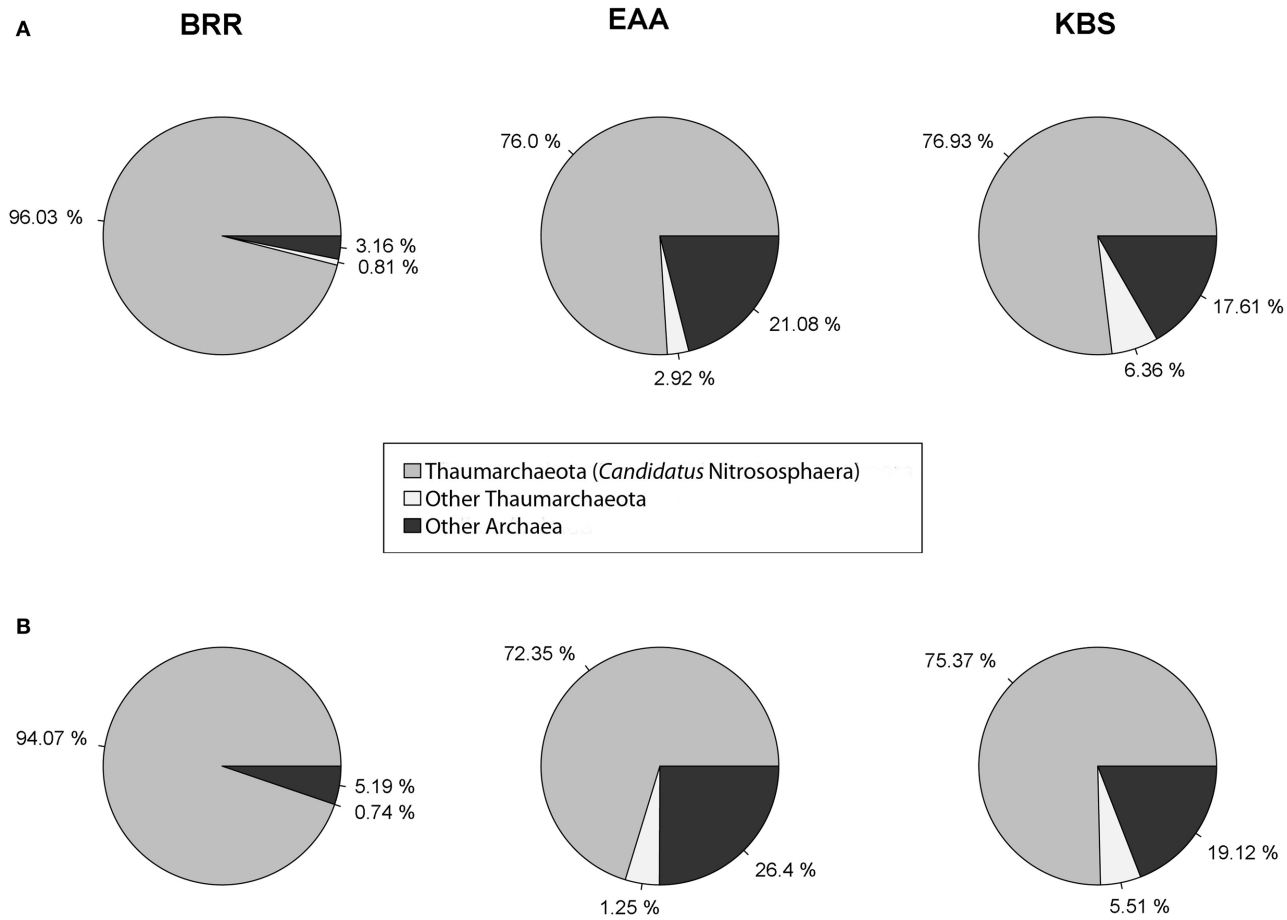
**Representation of total *Archaea* domain found in agricultural plots (A) and in non-agricultural plots (B) at the Broadbalk Experiment at Rothamsted Research (BRR), the Everglades Agricultural Area (EAA) and the Kellogg Biological Station (KBS)**.

The diversity of ammonia-oxidizing archaea (AOA) was examined at each site. Thaumarchaeota and *Ca.* Nitrososphaera were the most prevalent archaeal phylum and genus, respectively, at all sites and in agricultural and non-agricultural plots (Figure 4), showing a consistent pattern at all three sites. At BRR, *Ca.* Nitrososphaera comprised an average of 96% (agricultural plots) and 94% (non-agricultural plots) of total archaeal reads, representing the highest relative abundance of Thaumarchaeota among all three sites. *Ca.* Nitrososphaera comprised 76% (agricultural plots) and 72% (non-agricultural plots) of total Archaea for EAA and 77% (agricultural plots) and 75% (non-agricultural plots) of total Archaea for KBS. Other AOA, *Nitrosopumilus* and *Ca.* Nitrosocaldus were found in agricultural soils in very low abundance.

**Figure 5:**
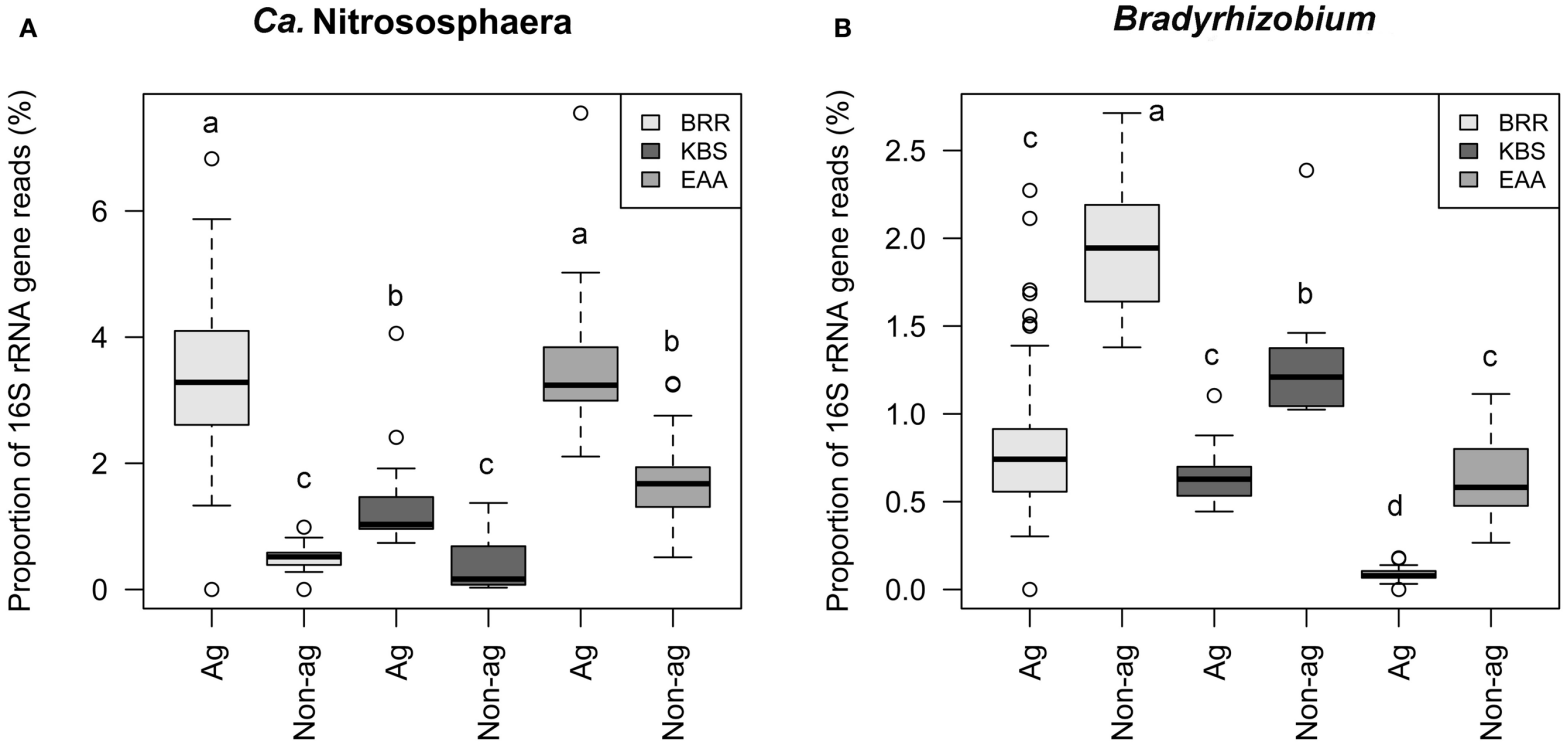
**Relative abundance of *Ca*. Nitrososphaera (A) and *Bradyrhizobium* (B) in soils at the Broadbalk Experiment at Rothamsted Research (BRR), the Everglades Agricultural Area (EAA) and the Kellogg Biological Station (KBS), separated into agricultural (Ag) and non-agricultural (Non-ag) plots.** The rectangular box represents the 25–75th percentiles, the whiskers represent the 10–90th percentiles, the line inside the box represents the median, and the open circles represent the outliers. The same letter above the box indicates no significant difference between means by Duncan's test at the 95% confidence interval.

The relative abundances of *Ca*. Nitrososphaera 16S rRNA genes were compared in agricultural and non-agricultural plots at the three study sites (Figure 5A). At all sites the relative abundances of *Ca*. Nitrososphaera were significantly higher in agricultural than in non-agricultural plots (Figure 5A). The relative abundance of this genus has increased twofold with agriculture at EAA and threefold at KBS. The BRR agricultural plots had the highest sevenfold increase in the relative abundance of the *Ca*. Nitrososphaera (*p* ≤ 0.001) compared to the non-agricultural unfertilized grassland and woodland plots. Prior to the start of the BRR experiment in 1843, this site had been in cultivation for at least 200 years (Powlson et al., 1986) and probably even longer (the Rothamsted estate map from 1623 shows the site as arable). Therefore, the decrease in the relative abundance of archaea in the non-agricultural plots has probably occurred within the last 125 years since cultivation ceased.

### *Bradyrhizobium* in agricultural and non-agricultural sites

The proportion of *Bradyrhizobium* 16S rRNA gene reads was higher at all three sites on non-agricultural compared to agricultural plots (Figure 5B). The highest abundance of *Bradyrhizobium* was found at BRR non-agricultural plots followed by the non-agricultural EAA plots. The relative abundance of this genus lowered with agriculture by two and threefold at BRR and KBS, respectively, and sevenfold at EAA compared to non-agricultural plots.

### Soil properties

At all three locations, soil pH was significantly higher in agricultural plots, which were slightly basic at BRR and EAA. pH was lower in non-agricultural plots, which were slightly acidic (Table 4). The soil organic matter (SOM) was significantly higher in all non-agricultural plots as compared to the agricultural plots (Table 4). The average moisture content at BRR was higher in non-agricultural plots, but at EAA, the moisture level was higher in soils under cultivation. The total soil nitrogen was higher in non-agricultural plots at BRR, whereas KBS had more nitrogen in agricultural plots. NH_3_ content was significantly higher in agricultural plots for BRR and EAA, but at KBS the level of NH_3_ did not change with agricultural management due to low pH.

**Table 4:** **Soil properties of agricultural and non-agricultural soils at Broadbalk Experiment at Rothamsted Research (BRR), the Everglades Agricultural Area (EAA) and the Kellogg Biological Station (KBS)**.

**Plot**	**pH**	**Moisture %**	**Organic matter %**	**Total N %**	**NO**^**−**^_**3**_**-N mg kg**^**−1**^	**NH**^**+**^_**4**_**-N mg kg**^**−1**^	**log[NH**_**3**_**]**
**BRR**							
Agricultural	^**^7.37	16.32	2.62	0.15	9.16	1.86	^**^−2.21
Non-agricultural	6.24	^*^24.08	^**^8.28	^**^0.41	6.14	1.29	−2.99
**EAA**							
Agricultural	^**^7.98	^*^122.64	71.10	ND	53.64	8.84	^**^−0.44
Non-agricultural	5.63	102.07	^*^83.31	ND	88.81	9.63	−2.85
**KBS**							
Agricultural	^**^5.81	ND	1.49	^*^0.13	^**^9.57	1.3	−3.32
Non-agricultural	5.2	ND	^**^2.43	0.09	1.38	^**^6.18	−3.24

* Significant, p-value ≤ 0.05

** Significant, p-value ≤ 0.001

ND-not determined.

### Correlations between soil parameters, *Ca.* nitrososphaera and *Bradyrhizobium*

**Figure 6:**
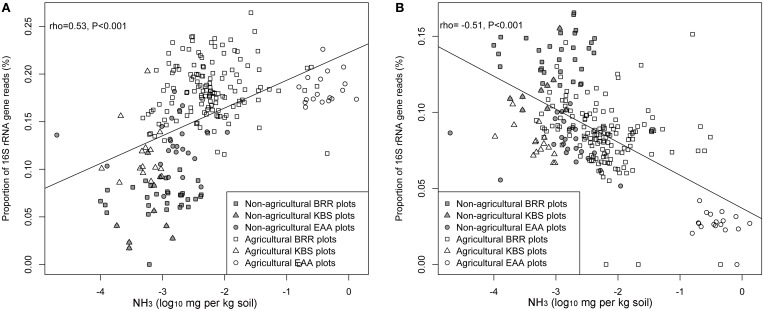
**Relationship between the proportion of *Ca.* Nitrososphaera 16S rRNA gene reads (%) and NH_3_ concentration (A); *Bradyrhizobium* and NH_3_ concentration (B) at three experimental sites.** Proportion of 16S rRNA reads was normalized by arcsine square root.

**Figure 7:**
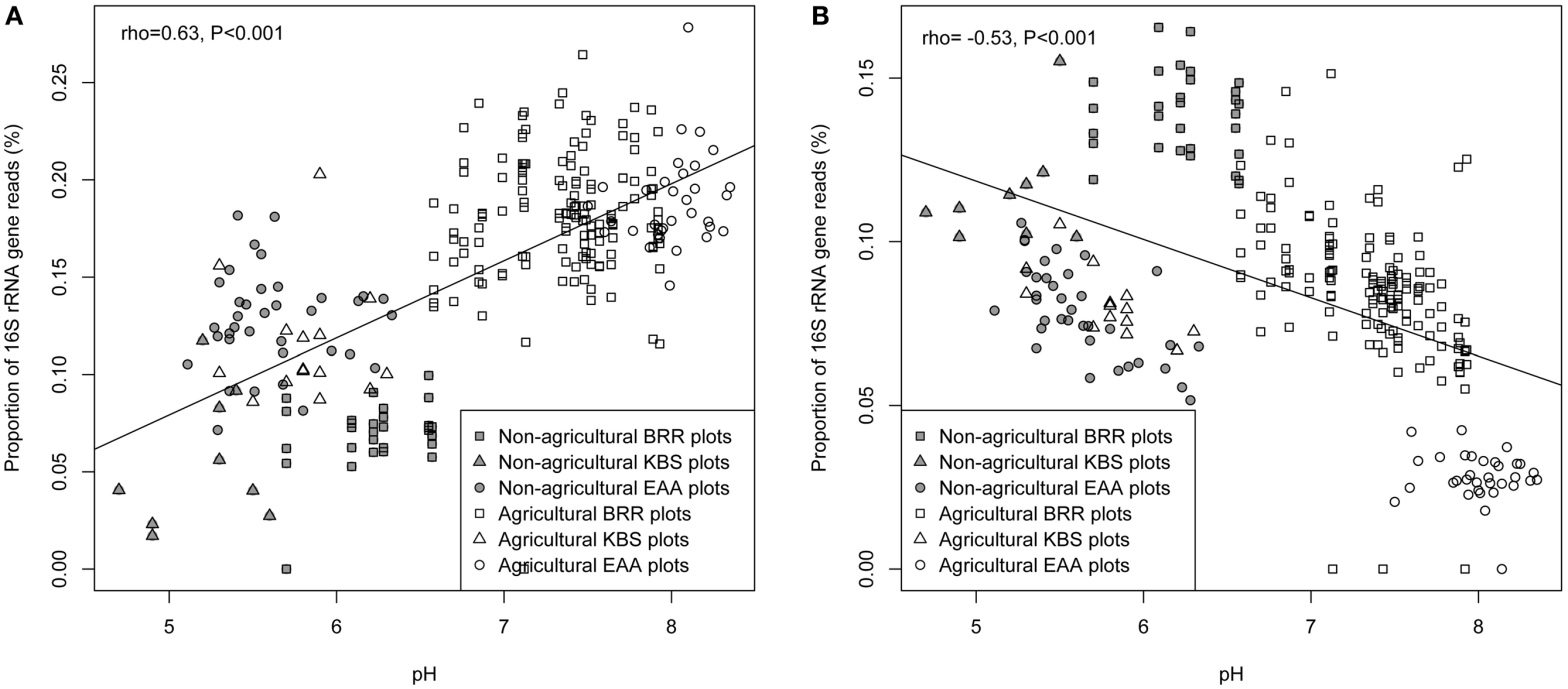
**Relationship between the proportion of *Ca.* Nitrososphaera 16S rRNA gene reads (%) and pH (A); *Bradyrhizobium* and pH (B) at three experimental sites.** Proportion of 16S rRNA reads was normalized by arcsine square root.

The relationships between each measured soil parameter and the relative abundance of *Ca.* Nitrososphaera at the three sites were determined by using Spearman correlation (Table 5). NH^+^_4_, SOM, total N, and moisture were significantly negatively correlated with the abundance of *Ca.* Nitrososphaera. However, NH_3_ and pH had the most significant and highest positive correlation with the relative abundance of this genus (Figures 6A and 7A). Moreover, at all sites, the relationships between the *Ca.* Nitrososphaera and either pH or NH_3_, were stronger in agricultural plots (Figures 6A and 7A).

**Table 5:** **Spearman correlation (rho) for Broadbalk experiment at Rothamsted research (BRR), the Everglades Agricultural Area (EAA) and the Kellogg Biological Station (KBS) between relative abundance of *Ca.* Nitrososphaera, *Bradyrhizobium*and soil features (TN, total nitrogen; NH^+^_4_, ammonium; NH_3_, ammonia; NO^−^_3_, nitrate; Moisture; SOM, organic matter and pH)**.

**Variables**	***Ca*. Nitrososphaera**	***Bradyrhizobium***
pH	^*^0.63	^*^−0.53
NH^+^_4_	^*^−0.27	−0.03
NH_3_	^*^0.53	^*^−0.51
SOM	^*^−0.24	−0.09
TN	^*^−0.37	^*^0.42
NO^−^_3_	−0.08	^*^−0.21
Moisture	^*^−0.41	^*^−0.24

Agricultural and non-agricultural plots were analyzed together (n = 158).

* Significant, p-value ≤ 0.001.

*Bradyrhizobium* was strongly negatively correlated with pH and the level of NH_3_ in soil (Figures 6B and 7B). Moisture and NO^−^_3_ also were significantly negatively correlated with *Bradyrhizobium*, but their effect was less negative than the effect of NH_3_ and pH level (Table 5).

No correlation was observed between the relative abundance of *Ca*. Nitrososphaera and *Bradyrhizobium* over the course of 13 months of monthly measurements at the Everglades Agricultural Area. Similarly, the differences in these two genera in agricultural and non-agricultural areas remained the same over these 13 months. At Broadbalk, the proportion of *Ca*. Nitrososphaera to *Bradyrhizobium* did not change during 5 months of monthly measurements in the agricultural plots. However, in the non-agricultural plots at Broadbalk, there was a statistically significant positive correlation across time in the proportion of *Ca*. Nitrososphaera to *Bradyrhizobium*(*r*^2^ = 0.252, *p* = 0.004).

## Discussion

No previous work shows the dramatic effects of land use management on soil microbial diversity on adjacent plots using long-term field experiments. In addition, the observation that these effects are reversible, as seen through the study of the Kellogg successional plots is also novel. In addition, no previous work identifies specific markers of land use change as has been done here with the discovery of *Ca*. Nitrososphaera as an abundant organism in agricultural soils and *Bradyrhizobium* as an abundant organism in non-agricultural soils and that these land use markers are reproducible across a suite of diverse sites and highly statistically significant. And no previous work has shown that ammonia is the driver of archaeal relative abundance in soils. These major findings are discussed below in the context of previous work.

This work was inspired by the results of Roesch et al. (2007) who found a high relative abundance of archaea in three agricultural soils (4–12%) and a vanishingly low number in a boreal forest soil (0.01%). This result led to several questions. Why were the archaea so low in the boreal compared to the others sites? Was it the colder climate in the boreal forest site from northern Ontario, Canada compared to the other sites? Was it the likely lower nutrient status of the forest site compared to the agricultural sites? Just prior to the publication of Roesch et al. (2007), the discovery of ammonia-oxidizing archaea in soil was made (Leininger et al., 2006). It was reasonable to assume that nitrogen fertilization of agricultural sites might contribute to the higher relative abundance of archaea in agricultural soils. To test this notion, three long-term experimental sites were chosen that met the following three important criteria. First, the sites had to be long-term sites with the availability of nutrient and other environmental data. Second, the sites had to have adjacent plots that were cultivated and uncultivated. Third, collectively the sites had to differ significantly in mean annual temperature to be able to test a climate effect on soil archaea. Using these criteria, three sites were chosen: the Broadbalk experiment at Rothamsted Research in the UK, the Kellogg Biological Station in Michigan USA, and the Everglades Agricultural Area in South Florida, USA. The Broadbalk and Kellogg sites had the added advantage of having experimental plots with varying amounts of N fertilizer applied annually.

In addition to the archaea, bacterial taxa were also examined for their changes with land use. Only about 24% of all reads could be classified to known genera. Of the more than 700 known genera found at each site, an average of 20 genera at each site were of reasonably high abundance that also differed in relative abundance between agricultural and non-agricultural plots. Of those, only two differed in relative abundance by land use at all three sites. One of these, *Bradyrhizobium*, is involved in ammonia production and is best known for its role as a nitrogen-fixing symbiont on legume roots. The other, *Ca*. Nitrososphaera, is best known for its role in ammonia oxidation. The two genera, *Bradyrhizobium* and *Ca*. Nitrososphaera, are negatively and positively correlated with ammonia levels, respectively. This also makes sense biochemically. Addition of fixed N inhibits nitrogenase and nitrogen fixation in the lab and field (Sekhon et al., 1987; Peoples et al., 1995; Halbleib and Ludden, 2000). In addition, as the product of nitrogenase, ammonia is a product inhibitor of nitrogenase and also blocks transcription of the *nif*regulon (Halbleib and Ludden, 2000). As a result, higher levels of N would reduce the role for nitrogen-fixing organisms in non-legume agriculture at each of the three sites studied here. The observation that the proportion of *Ca*. Nitrososphaera to *Bradyrhizobium* increases slightly, but significantly statistically, over time at Broadbalk supports the notion that *Ca*. Nitrososphaera's relative numbers compared to *Bradyrhizobium* increase with increasing ammonia concentrations in soil. This may occur because *Bradyrhizobium* is providing more ammonia to soil, which encourages a population increase among the ammonia oxidizing archaea in a non-agricultural soil. In an agricultural soil where N fertilizer is applied, the number of free-living bradyrhiozbia declines since fixed N inhibits N_2_ fixation. So the numbers of ammonia oxidizing archaea increase in an agricultural soil as a result of added N and a higher soil pH. Both factors result in the increased availability of ammonia.

The above hypothesis depends on nitrogen fixation by free-living *Bradyrhizobium* in soil since the samples collected in this work were from soil and did not contain roots. There are several examples of free-living nitrogen fixation by *Bradyrhizobium* (formerly referred to as slow-growing *Rhizobium*). This was first shown by Pagan et al. (1975) who demonstrated free-living nitrogen fixation by strain 32H1 as well as other strains. This work was followed shortly by experiments that optimized the conditions for nitrogenase activity by free-living bradyrhizobia including O_2_ requirements as well as the cell morphology changes that occur under nitrogen fixation conditions (Gibson et al., 1976; Keister and Evans, 1976; van Brussel et al., 1979). These observations were soon expanded to still more strains of *Bradyrhizobium* (Subba-Rao, 1977; Skotnicki et al., 1979). Nevertheless, attempts to obtain free-living nitrogen fixation in many bradyrhizobia have failed (Pagan et al., 1975; Skotnicki et al., 1979). However, this may be caused by the lack of a specific nutrient in medium. For example, it was recently shown that symbiotic rhizobia lack *nifV*, a gene essential for the production of homocitrate, a necessary component of the FeMo cofactor present in dinitrogenase and that the host plant provides homocitrate to the nodule bacteria to compensate for the lack of *nifV* (Hakoyama et al., 2009).

Nevertheless, free-living bradyrhizobia have been shown to fix ^15^N_2_ in soil using stable isotope probing. ^15^N label was found in bradyrhizobial 16S rRNA sequences after feeding ^15^N_2_ to soil mesocosms (Buckley et al., 2007). In addition the genome of a free-living *Bradyrhizobium* strains isolated from a rice paddy was recently sequenced and found to contain the same complement of nitrogen fixation genes found in the genome of a nitrogen-fixing symbiont of *Bradyrhizobium*. However, this paddy soil *Bradyrhizobium* strain did not nodulate any legume tested, lacked a symbiosis island of genes often found in N_2_-fixing legume symbionts, and did not possess any of the nodulation genes. All of these results taken together with the results presented here are expected to encourage an examination of free-living bradyrhizobia in uncultivated soils to determine their ability to provide fixed N to unmanaged ecosystems.

There is also a sound biological basis for *Ca*. Nitrososphaera to be relatively more abundant in agricultural soils than non-agricultural soils. As ammonia, not ammonium, is the substrate for ammonia monooxygenase (Suzuki et al., 1974; Arp et al., 2002), it is not surprising that ammonia levels, not ammonia plus amonium levels, correlate well with the relative abundance of *Ca*. Nitrososphaera, particularly at higher pH levels. However, discovering this required that ammonia levels be calculated from the total ammonia plus ammonium levels and soil pH as current methods of measuring ammonia/ammonium levels in soil do not distinguish between ionized ammonium and non-ionized ammonia.

## Conclusion

The results here show that agricultural management causes significant changes in soil, which leads to an increase in AOA abundance. *Ca*. Nitrososphaera, the most abundant soil AOA, was present in a greater abundance at all three sites in response to agriculture. Of all factors examined, pH mediated NH_3_ accumulation was the primary driver of the AOA community in soil.

In addition, this work shows the effect of agriculture on the relative abundance of other organisms involved in the nitrogen cycle. At each site, the relative abundance of *Bradyrhizobium*, a nitrogen-fixing symbiont, was strongly negatively correlated with agricultural land use, pH, and NH_3_ levels. The reciprocal responses of *Bradyrhizobium* and *Ca*. Nitrososphaera appear to be excellent biological markers for land use. For further validation of these microorganisms as biological markers, these results should encourage the testing of these genera as markers for land use at other sites.

## Accession numbers

All sequences have been deposited in the GenBank database with Accession No. PRJNA191521, RJNA191098, and PRJNA191523.

### Conflict of interest statement

The authors declare that the research was conducted in the absence of any commercial or financial relationships that could be construed as a potential conflict of interest.
